# Maternal environmental, occupational, and urinary metabolite levels of benzene compounds and their association with congenital heart diseases in offspring: a case‒control study in China

**DOI:** 10.1007/s11356-023-27015-z

**Published:** 2023-04-25

**Authors:** Meixian Wang, Lu Li, Hong Kang, Hongmei Xu, Qian Huang, Nana Li, Ying Deng, Ping Yu, Zhen Liu

**Affiliations:** 1 Key Laboratory of Birth Defects and Related Diseases of Women and Children, Ministry of Education, National Office for Maternal and Child Health Surveillance of China, West China Second University Hospital, Sichuan University, Chengdu, 610041 China; 2 Department of Gynaecology and Obstetrics, Leshan People’s Hospital, Leshan, 614003 China; 3 Department of Gynaecology and Obstetrics, Shehong People’s Hospital, Shehong, 629299 China

**Keywords:** Congenital heart defects, Environmental factors, Maternal occupation, Benzene compound metabolites, Pregnancy

## Abstract

The conclusions about the association of maternal pregnancy environment, occupation, and benzene compounds with fetal CHD are not entirely consistent. Eight hundred seven CHD cases and 1008 controls were included in this study. All occupations were classified and coded against the Occupational Classification Dictionary of the People’s Republic of China (2015 version). Logistic regressions were used to explore the correlation among environmental factors, occupation types, and CHDs in offspring. We found that living near public facilities and having exposure to chemical reagents and hazardous substances were significant risk factors for CHDs in offspring. We found that offspring of mothers who worked in agriculture and similar work during pregnancy suffered from CHD. The risk of all CHDs in the offspring of pregnant women working in production manufacturing and related work was significantly higher than that in unemployed pregnant women, the risk was also observed in 4 subtypes of CHDs. We compared the concentrations of the five metabolite (MA, mHA, HA, PGA, and SPMA) levels of benzene compounds in the urine of mothers in case and control groups and found no significant differences. Our study suggests that maternal exposure during pregnancy and certain environmental and occupational conditions are risk factors for CHD in offspring, but did not support an association between concentrations of metabolites of benzene compounds in the urine of pregnant women and CHDs in their offspring.

**Supplementary Information:**

The online version contains supplementary material available at 10.1007/s11356-023-27015-z.

## Introduction

Congenital heart defects (CHDs) are the most common congenital malformation of all birth defects, accounting for almost 28% of major birth defects (Dolk et al. 2011; van der Linde et al. 2011). CHDs are rich in variety and complex in etiology. However, despite many studies that have contributed significantly to the study of CHD etiology, it has been shown that only one third of CHD cases have been shown to be caused simply by genes. There is greater agreement that CHDs can also be caused by genetic factors, environmental factors, and/or gene‒environment interactions (Kalisch-Smith et al. 2020). The reported prevalence of CHDs continues to increase globally. CHDs are the most common malformations in newborns. The global prevalence of CHDs increased 10% every 5 years from 1970 to 2017 in Asia (Liu et al. 2019). Accordingly, evaluating environmental determinants for CHDs is truly required, because in contrast with hereditary elements, environmental factors are modifiable.

All people are exposed to a variety of adverse environmental factors. For example, polycyclic aromatic hydrocarbons (PAHs), PM2.5, and metals in the atmosphere, water, and soil are a risk to human health (Ambade et al. 2022, 2023; Kumar et al. 2020; Sun et al. 2022; Vithanage et al. 2022). Maternal exposure during pregnancy is important for the health of the fetus. There have been a number of studies on maternal environmental exposures during pregnancy and fetal CHDs, including occupational exposures. Hazardous substances from environmental or occupational exposure, and organic solvent exposure have all been shown to be potential risk factors for CHDs (Gorini et al. 2014; Kalisch-Smith et al. 2020). Maternal exposure to environmental toxins during pregnancy can increase the risk of CHDs in offspring (Cresci et al. 2013). The living environment during pregnancy is also very important. Pregnant women living near chemical plants and coal plants have an increased risk of CHDs in their offspring (Viglianco et al. 2019). And prolonged exposure to high temperatures during pregnancy may increase the risk of ventricular septal defects and atrial septal defects in their offspring (Lin et al. 2018; Zhang et al. 2019).

Occupational exposure is an important environmental exposure for pregnant women who continue to work during pregnancy, as they spend long hours in the work environment. Increasingly, women may face occupational hazards at work due to exposure to organic solvents, pollutants, toxins, or contaminated water (Rosman et al. 2020). Working in certain occupations during pregnancy may affect pregnancy outcomes. Food industry workers have an increased risk of preterm birth (Casas et al. 2015). Occupational exposure to inorganic particles or welding fumes during pregnancy is associated with negative birth outcomes (Norlén et al. 2019). Maternal occupation is also associated with birth defects. For example, some occupational groups are positively associated with one or more birth defects, including janitors/cleaners, scientists, and electronic equipment operators (Herdt-Losavio et al. 2009; Siegel et al. 2019). Working in certain jobs increased the risks of several malformations, such as maids, janitors, biologists, chemists, etc. (Lin et al. 2013). Previous studies have demonstrated the adverse effects of specific toxicants, such as metals and organic solvents, including benzene and toluene, on pregnancy and birth outcomes (Kumar et al. 2019). In addition, some occupational activities, such as weight lifting, prolonged standing, walking, and bending, increased the risk of adverse outcomes in pregnancy (Cai et al. 2020). Occupational exposure to hazardous substances is very dangerous for fetal heart development. There is an association between maternal work as a nail technician during pregnancy and fetal CHDs (Siegel et al. 2021). Mothers who work in the garment manufacturing industry have an increased risk of CHDs in offspring, mainly due to oil mist exposure at work (Siegel et al. 2019).

A few studies have evaluated the association between benzene and nonisolated pulmonary atresia (Tanner et al. 2015), cleft palate (Ramakrishnan et al. 2013), and major congenital, and neural crest malformations (Janitz et al. 2018; Wennborg et al. 2005). Some studies have shown that exposure to a mixture of benzene, toluene, ethylbenzene, and xylene (BTEX) compounds during pregnancy may lead to premature birth (PTB) in pregnant women (Cassidy-Bushrow et al. 2021a; Santos and Nascimento 2019). Frequent exposure to or the use of aromatic solvents (e.g., o-xylene, styrene, and ethylbenzene) during pregnancy may have toxic vestibular effects on the fetus (Tallandier et al. 2021). According to the results of our literature review, only a small number of studies have evaluated the association between BTEX compounds and CHDs.

The existing studies about occupational exposure and CHDs are mainly on high-risk or specific types of occupations. We found no studies assessing the association between various types of occupations and CHDs in Chinese pregnant women. Therefore, we conducted a case‒control study in China with multiple hospital participants based on the Chinese National Birth Defects Surveillance System. This study used a detailed questionnaire interview method to assess environmental and occupational exposures of pregnant women in the peri-pregnancy period and to collect urine biological samples. This study aimed to assess the association between periconceptional environmental exposure, particularly occupational exposure, and CHDs in offspring. After completing the assessment, we further explored whether major exposure to benzene and its derivatives is involved in the association.

## Methods

### Study design

This was a multicenter study that included six tertiary care hospitals, and participants in this study were recruited from among pregnant women who underwent prenatal screening at these institutions. These participating hospitals were all qualified for prenatal diagnosis and were included from February 2010 to August 2014. We recruited the pregnant women whose fetuses were diagnosed as having CHDs by echocardiography, and a control group of pregnant women whose fetuses had not been diagnosed without any abnormality. The selection criteria for the control group were that they were from the same study period and hospital as the case group, and had a gestational age difference of no less than 2 weeks (Wang et al. 2022). Once a pregnant woman was included in case group, the first one or two voluntary pregnant women who meet the control group selection criteria was chosen as the proper control.

There were strict exclusion criteria for participation in the study. Pregnant women with multiple fetuses, a gestational age less than 14 weeks, fetuses with other noncardiac malformations, fetuses with chromosomal diseases or monogenic disorders, and fetuses with unclear diagnoses were excluded from the study. Of course, pregnant women with mental disorders were not included in recruitment.

### Questionnaire interview

All participating pregnant women recruited for this study were interviewed face-to-face during their prenatal visits. The questionnaire collected information in three parts. (1) Basic maternal information, including maternal age, height, and weight (used to calculate body mass index, BMI), education level, and place of residence at the time of recruitment. (2) Nutritional supplements and lifestyle, including whether women took folic acid before 12 weeks of pregnancy, whether they were exposed to cigarette smoke (including maternal smoking and exposure to secondhand smoke in the surrounding environment after becoming pregnant), and whether they consumed alcohol (refers to whether the pregnant woman had consumed alcohol after becoming pregnant). (3) Environmental and occupational exposures from the first trimester to the time of the interview, including whether the home had been renovated, whether there were public facilities near the home (including large landfills, garbage houses, large waste incinerators, large highways or traffic arteries), whether there was exposure to chemical agents (including organic materials, disinfectants, and organic solvents), whether there was regular exposure to hazardous substances (including metal agents, harmful solids, harmful gases, and harmful organisms), and occupational information (the longest job since becoming pregnant). All of the above information was self-reported by the pregnant women.

### Occupational classification codes

In this study, it was specified that work performed from the first 3 months of pregnancy until the time of the interview was considered as the exposure. All the occupations of the pregnant women included in this study were classified and coded against the standard classification of the Occupational Classification Dictionary of the People’s Republic of China (2015 version). The occupations of the participants in this study included 5 major categories: (a) professional and technical staff, the code is 2 (GBM 20000); (b) clerical and related personnel, the code is 3 (GBM 30000); (c) social production service and life service personnel, the code is 4 (GBM 40000); (d) agriculture, forestry, animal husbandry, fishery production and supporting personnel, the code is 5 (GBM 50000); and (e) production manufacturing and related personnel, the code is 6 (GBM 60000).

### Determination and classification of CHDs

The diagnosis of birth defects in this study was very strict to ensure the accuracy of the diagnosis. For live births, the diagnosis was confirmed by a combination of ultrasound, obstetric specialists and pediatric specialists, and the fetuses of participants with stillbirths and miscarriages were diagnosed by autopsy. Additionally, 4 ~ 5 national prenatal ultrasound specialists and pediatric cardiologists reviewed the echocardiographic images of all CHD cases. The classification of CHDs referred to existing studies and classified CHD cases into six major categories based on anatomical lesions and clinical staging: atrial septal defects (subgroup 1); coronary artery defects (subgroup 2); left ventricular outflow tract obstructions (subgroup 3); right ventricular outflow tract obstructions (subgroup 4); abnormal pulmonary venous return (subgroup 5); and other cardiac defects (subgroup 6) (Malik et al. 2008).

### Urine sample collection and chemical concentration measurements

Clean midstream urine was collected from pregnant women after the interview, and all urine samples were stored in labeled sterile lyophilized tubes and placed in a −80 °C refrigerator until use. Because it involved the collection of questionnaire information and biospecimens, this study was approved by the Medical Ethics Committee of Sichuan University (No. 2010004). All participants in this study signed an informed consent form. In this study, 835 (82.8%) and 390 (48.3%) participants’ urine samples were collected in the control and case groups. Due to the study funding constraints, we randomly sampled 40% of the samples in each of the two groups to measure benzene metabolite. Before the start of the assay, urine samples were thawed and their creatinine concentrations were measured. Urine samples with creatinine concentrations consistent with 0.3 to 3.0 g/L were used for the next assay, and the rest were discarded. Only 476 samples (323 controls and 153 cases) were available to measure benzene metabolites.

Major exposure to benzene and its derivatives in this study included benzene, toluene, ethylbenzene, xylene, and styrene. The determination of S-phenylmercapturic acid (SPMA) in urine has been proposed as a suitable biomarker for monitoring of low-level exposures to benzene (Boogaard and van Sittert 1996, van Sittert et al. 1993). Information on toluene and xylene exposure can be provided by the concentration of hippuric acid (HA) and methylhippuric acid (mHA) in the human urine matrix (Shan et al. 2021). Mandelic acid (MA) and phenylglyoxylic acid (PGA) are suitable biological markers of ethylbenzene and styrene exposure (Capella et al. 2019).

The concentrations of SPMA, MA, mHA, HA, and PGA were measured in urine by an Acquity UPLC (Waters, USA) with a 3200 Q TRAP System (AB SCIEX, USA). The experimental method was based on the method used by Zhu et al. (Zhu et al. 2022). The urine sample was centrifuged at 15000 rpm for 5 min and diluted 10 times with 15 mmol/L ammonium acetate buffer (the buffer contained 50 ng/mL of each isotopic internal standard of the analyte to be measured), and the sample was infused into the HPLC‒MS/MS for determination. The chromatographic partition was performed on a C_18_ Sect. (1.7 µm × 2.1 mm × 50 mm waters, USA) with slope elution. Ionization was directed with electrospray ionization in regrettable mode. The MS/MS particle source working circumstances were set as follows: a shower voltage of − 4400 V; sheath gas, helper gas, and drape gas (nitrogen) pressures of 30, 45, and 50 psi; and a vaporizer temperature of 400 °C. All concentration values were corrected for creatinine.

### Statistical analysis

In this study, the results were described by the median (interquartile variance) for skewed data and by number (%) for grouped data. The Mann‒Whitney *U* test and *χ*^2^ test were used to compare the participant characteristics of the case and control groups. Binary and multivariate logistic regression were used to explore the relationship among environmental factors, type of occupation, and CHDs in offspring. The selection of covariates to be included in the logistic regression model was based on two main principles: (1) these factors are statistically relevant to the results in this analysis, or (2) they may be related to both exposure and outcome based on previous studies.

The data of SPMA, MA, mHA, HA, and PGA concentrations in urine were identified to be nonnormally distributed and are described using box plots. Differences in concentrations between the control group and all CHD groups and CHD subgroups were determined using the Mann‒Whitney *U* test. Differences in concentrations between different occupational groups were determined using the Kruskal‒Wallis *H* test.

The statistical analysis software used for this study was SPSS v.25.0 (SPSS Inc., Chicago: IL) with a significance level of 0.05.

## Results

### Characteristics of participants

After strict inclusion and exclusion criteria screening, 807 CHD cases and 1008 controls were included in this study. The case group included 293 fetuses (36.31%) with conotruncal defects, 269 (33.33%) with septal defects, 99 (12.27%) with right ventricular outflow tract obstructions, 79 (9.79%) with left ventricular outflow tract obstructions, 34 (4.21%) with anomalous pulmonary venous return, and 33 (4.09%) with other heart defects. Maternal age, maternal BMI, maternal education level, taking folic acid before 12 weeks of pregnancy, maternal drinking, living in urban or rural areas, and city were significantly different between the case and control groups. The case group had a higher proportion of pregnant women with low education levels, a lower proportion of women who took folic acid before 12 weeks of pregnancy, and a higher proportion of women living in rural areas. However, there was no difference in cigarette smoke exposure between the two groups (see Table 1).

**Table 1:** The maternal characteristics of case and control participants

Variable	Controls (*n* = 1008)	Cases (*n* = 807)	*P* value^a^
Maternal age			
Median (interquartile range)	28 (6)	28 (6)	0.001^*^
Maternal BMI			
Median (interquartile range)	19.95 (3.10)	19.56 (3.05)	0.033^*^
Maternal education			
Junior high school and below	160 (15.87)	258 (31.97)	< 0.001^*^
High school secondary school	243 (24.11)	191 (23.67)	
College and above	576 (57.14)	311 (38.54)	
Missing	29 (2.88)	47 (5.82)	
Folic acid taken before 12 weeks			
Yes	777 (77.08)	533 (66.05)	< 0.001^*^
No	157 (15.58)	179 (22.18)	
Missing	74 (7.34)	95 (11.77)	
Cigarette smoke exposure			
Yes	162 (16.07)	150 (15.59)	0.158
No	846 (83.93)	657 (81.41)	
Drinking			
Yes	258 (25.60)	138 (17.10)	< 0.001^*^
No	750 (74.40)	669 (82.90)	
Residence			
Urban	928 (92.06)	609 (75.46)	< 0.001^*^
Rural	52 (5.16)	164 (20.32)	
Missing	28 (2.78)	34 (4.21)	
City			
Shenzhen	440 (43.65)	244 (30.24)	< 0.001^*^
Fujian	391 (38.79)	87 (10.78)	
Guangxi	106 (10.52)	217 (26.89)	
Hubei	59 (5.85)	112 (13.88)	
Sichuan	7 (0.69)	121 (14.99)	
Xi'an	5 (0.50)	26 (3.22)	

^*^Significant results, *P* value < 0.05

^a^Calculated by Mann–Whitney *U* test for maternal age, maternal BMI, the *χ*^2^ test for maternal education, folic acid taken before 12 weeks, cigarette smoke exposure, drinking, maternal residence, and city

### Living/working environment exposure

This study collected data on the exposure of these pregnant women to their living and working environments between the first trimester of pregnancy and the date of investigation. It was found that the proportion of pregnant women in the case group whose homes had been renovated in the last year, whose homes were near public facilities, and who were exposed to chemical reagents and hazardous substances was significantly higher than that in the control group. We explored the relationship between these exposures and CHDs using multivariate logistic regression models adjusted for maternal age, BMI, education level, taking folic acid before 12 weeks of pregnancy, drinking, residence, and city. We found that the offspring of pregnant women whose homes were near public facilities were more likely to have CHDs, and the adjusted odds ratio (aOR) was 1.40 (95% CI: 1.004,1.955). Periconceptional chemical reagent and hazardous substance exposure were also significant risk factors for CHDs in offspring, with aORs of 1.86 (95% CI: 1.283, 2.708) and 2.53 (95% CI: 1.741, 3.726) (see Table 2).

**Table 2:** Environmental exposure of case and control group

Environmental exposure	Controls, *n* (%)	Cases, *n* (%)	OR (95%CI)	aOR^a^ (95%CI)
House renovated				
Yes	123 (12.2)	145 (17.97)	1.576 (1.215, 2.045)^*^	1.325 (0.963,1.822)
No	885 (87.8)	662 (82.03)		
Public facilities near home ^b^				
Yes	110 (10.91)	127 (15.74)	1.525 (1.159, 2.005)^*^	1.401 (1.004,1.955)^*^
No	898 (89.09)	680 (84.26)		
Exposed to chemical reagents^c^				
Yes	96 (9.52)	104 (12.89)	1.405 (1.047, 1.886)^*^	1.864 (1.283,2.708)^*^
No	912 (90.48)	703 (87.11)		
Exposure to hazardous substances^d^				
Yes	71 (7.04)	112 (13.88)	2.127 (1.555, 2.909)^*^	2.527 (1.741,3.726)^*^
No	937 (92.96)	695 (86.12)		

^*^Significant results, *P* value < 0.05

^a^Adjusted for maternal age, BMI, education, folic acid taken before 12 weeks, drinking, residence, city

^b^The public facilities include large landfills, garbage houses, large waste incinerators, large roads, and traffic arteries

^c^The chemical reagents include organic raw materials, disinfectants, and organic solvents

^d^The hazardous substances include metal agents, harmful solids, harmful gases, and harmful organisms

### Different occupations and CHD risk

The survey results show that the largest number of pregnant women are social production service and life service personnel (*n* = 835), followed by clerical and related personnel (*n* = 289). There are 236 pregnant women whose occupation is professional and technical staff, and 107 pregnant women are production manufacturing and related personnel; 19 pregnant women are engaged as agriculture, forestry, animal husbandry, fishery production, and supporting personnel. In addition, there are 99 participants had unspecific job descriptions that could not be classified, and 230 pregnant women were not working or were unemployed. We also counted the number of middle category occupations under each major category (see Table S1 in supplementary materials).

We evaluated the risk of CHD and CHD subtypes in the offspring of mothers with different occupations during pregnancy. The data showed that all the offspring with CHDs had mothers who worked in agriculture, forestry, animal husbandry, fishery production, and supporting roles during pregnancy. A more detailed classification of the 19 pregnant women with occupation code 5 (GBM 50000) showed that they were mainly engaged in agricultural production, which suggests that maternal work in agricultural production during pregnancy is a very important risk factor for CHDs in fetuses (see Table 3).

**Table 3:** occupational type of control group, all CHDs group and six CHDs subgroups

Occupation code (major category)		2(GBM 20000)^a^	3(GBM 30000)^a^	4(GBM 40000)^a^	5(GBM 50000)^a^	6(GBM 60000)^a^	No coded^a^	Uncoded^a^
Controls	*n* (%)	153 (15.18)	182 (18.06)	492 (48.81)	0 (0)	30 (2.98)	112 (11.11)	39 (3.87)
All CHDs	*n* (%)	83 (10.29)	107 (13.26)	343 (42.5)	19 (2.35)	77 (9.54)	118 (14.62)	60 (7.43)
	aOR^b^ (95%CI)	0.795 (0.497,1.274)	0.981 (0.636,1.514)	0.663 (0.465,0.946)^*^	—^c^	2.023 (1.137,3.599)^*^	Ref	—^d^
Septal defects	*n* (%)	29 (10.78)	37 (13.75)	112 (41.64)	9 (3.35)	18 (6.69)	41 (15.24)	23 (8.55)
	aOR^b^ (95%CI)	0.792 (0.39,1.609)	1.174 (0.617,2.236)	0.664 (0.398,1.107)	—^c^	1.356 (0.575,3.2)	Ref	—^d^
Conotruncal defects	*n* (%)	27 (9.22)	35 (11.95)	126 (43)	6 (2.05)	29 (9.9)	46 (15.7)	24 (8.19)
	aOR^b^ (95%CI)	0.751 (0.382,1.475)	0.898 (0.477,1.692)	0.66 (0.403,1.079)	—^c^	2.476 (1.168,5.249)^*^	Ref	—^d^
Left ventricular outflow track obstruction	*n* (%)	14 (14.14)	13 (13.13)	43 (43.43)	3 (3.03)	12 (12.12)	11 (11.11)	3 (3.03)
	aOR^b^ (95%CI)	1.715 (0.602,4.884)	1.571 (0.606,4.069)	0.877 (0.396,1.942)	—^c^	3.018 (1.043,8.73)^*^	Ref	—^d^
Right ventricular outflow track obstruction	*n* (%)	5 (6.33)	9 (11.39)	39 (49.37)	1 (1.27)	7 (8.86)	11 (13.92)	7 (8.86)
	aOR^b^ (95%CI)	0.696 (0.233,2.077)	0.996 (0.369,2.688)	0.846 (0.394,1.82)	—^c^	4.37 (1.515,12.609)^*^	Ref	—^d^
Abnormal pulmonary venous return	*n* (%)	3 (8.82)	3 (8.82)	12 (35.29)	0 (0)	8 (23.53)	6 (17.65)	2 (5.88)
	aOR^b^ (95%CI)	0.789 (0.141,4.417)	0.506 (0.108,2.369)	0.639 (0.185,2.213)	—^c^	4.228 (1.049,17.047)^*^	Ref	—^d^
Other cardiac defect	*n* (%)	5 (15.15)	10 (30.3)	11 (33.33)	0 (0)	3 (9.09)	3 (9.09)	1 (3.03)
	aOR^b^ (95%CI)	0.802 (0.157,4.09)	1.592 (0.381,6.654)	0.441 (0.102,1.904)	—^c^	3.085 (0.451,21.105)	Ref	—^d^

^*^Significant results, *P* value < 0.05

^a^2(GBM 20000), professional and technical staff; 3(GBM 30000), clerical and related personnel; 4(GBM 40000), social production service and life service personnel; 5(GBM 50000), agriculture, forestry, animal husbandry, fishery production and supporting personnel; 6(GBM 60000), production manufacturing and related personnel; No coded, not working or unemployed; Uncoded, unspecified

^b^Adjusted for maternal age, BMI, education, folic acid taken before 12 weeks, drinking, residence, city

^c^The OR/aOR cannot be calculated because of the number of controls is 0

^d^We could not determine if there was a relevant exposure because the occupation was not specific, the group was not involved in the comparison

The risk of all CHDs in the offspring of pregnant women working in production manufacturing and related occupations (especially textiles, clothing and leather, and fur product processing and production) was significantly higher than that in unemployed pregnant women (aOR = 2.023, 95% CI: 1.137, 3.599). We further analyzed the association between pregnant women working in production manufacturing and related occupations and the six CHD subgroups. We found no significant risk for septal defects and other cardiac defects in offspring but found varying degrees of risk for conotruncal defects (aOR = 2.476, 95% CI: 1.168, 5.249), left ventricular outflow tract obstructions (aOR = 3.018, 95% CI: 1.043, 8.73), right ventricular outflow tract obstructions (aOR = 4.37, 95% CI: 1.515, 12.609), and abnormal pulmonary venous return (aOR = 4.228, 95% CI: 1.049, 17.047) in offspring (see Table 3).

### Concentration of benzene metabolites in maternal urine

Based on our findings in the analysis of the association between maternal occupation and CHDs, the offspring of mothers who work in production manufacturing and related positions or agriculture, forestry, animal husbandry, fishery production, and supporting roles have a higher risk for CHDs. We suspect may be due to benzene exposure at work. We measured the concentrations of MA, mHA, HA, PGA, and SPMA in the urine of 476 participants.

**Fig. 1:**
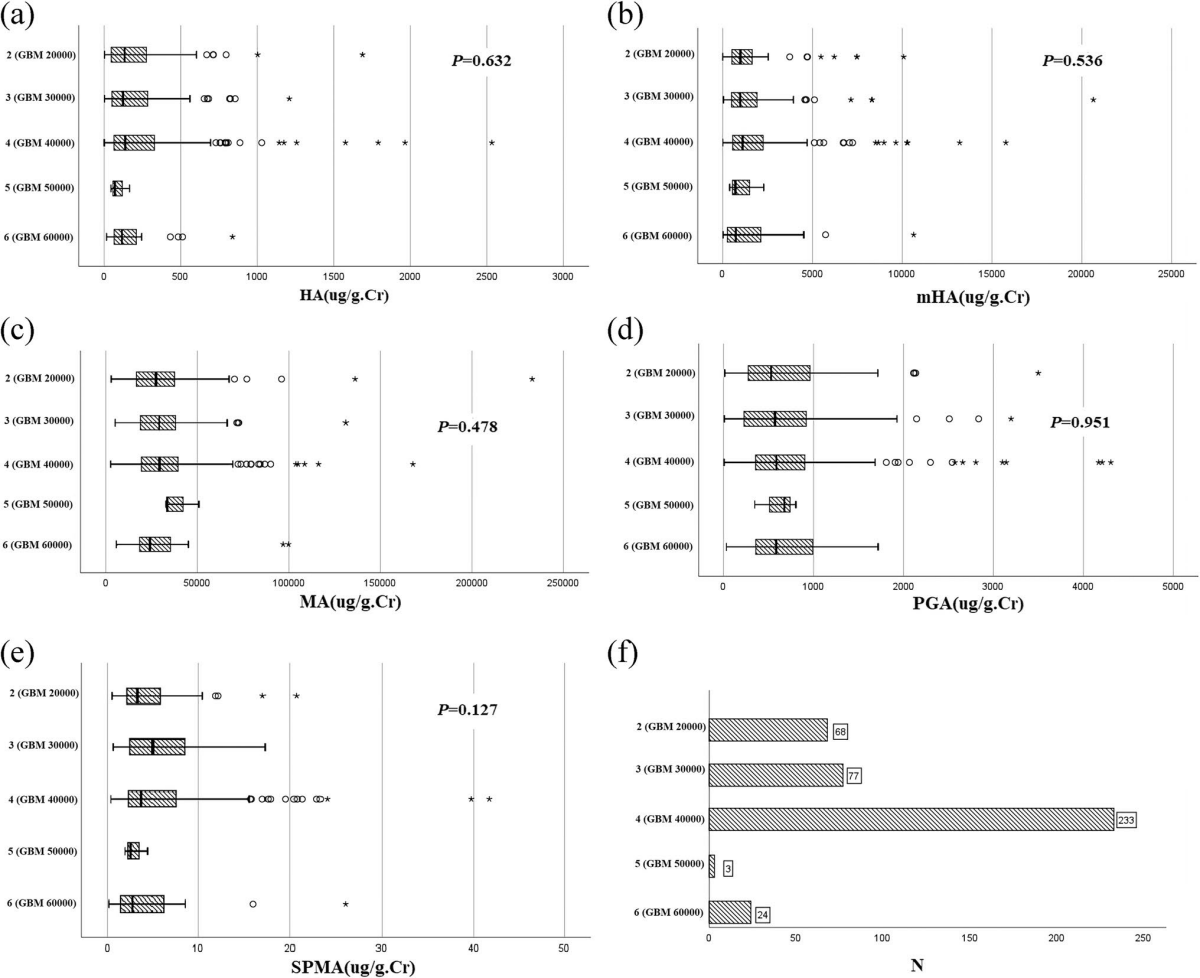
Concentrations of five urinary metabolites in different occupational types ("○" inside the image represents mildly abnormal values (outside of Q1-1.5 times IQR or Q3+1.5 times IQR) and "*" indicates extreme abnormal values (outside of Q1-3 times IQR or Q3+3 times IQR))

**Fig. 2:**
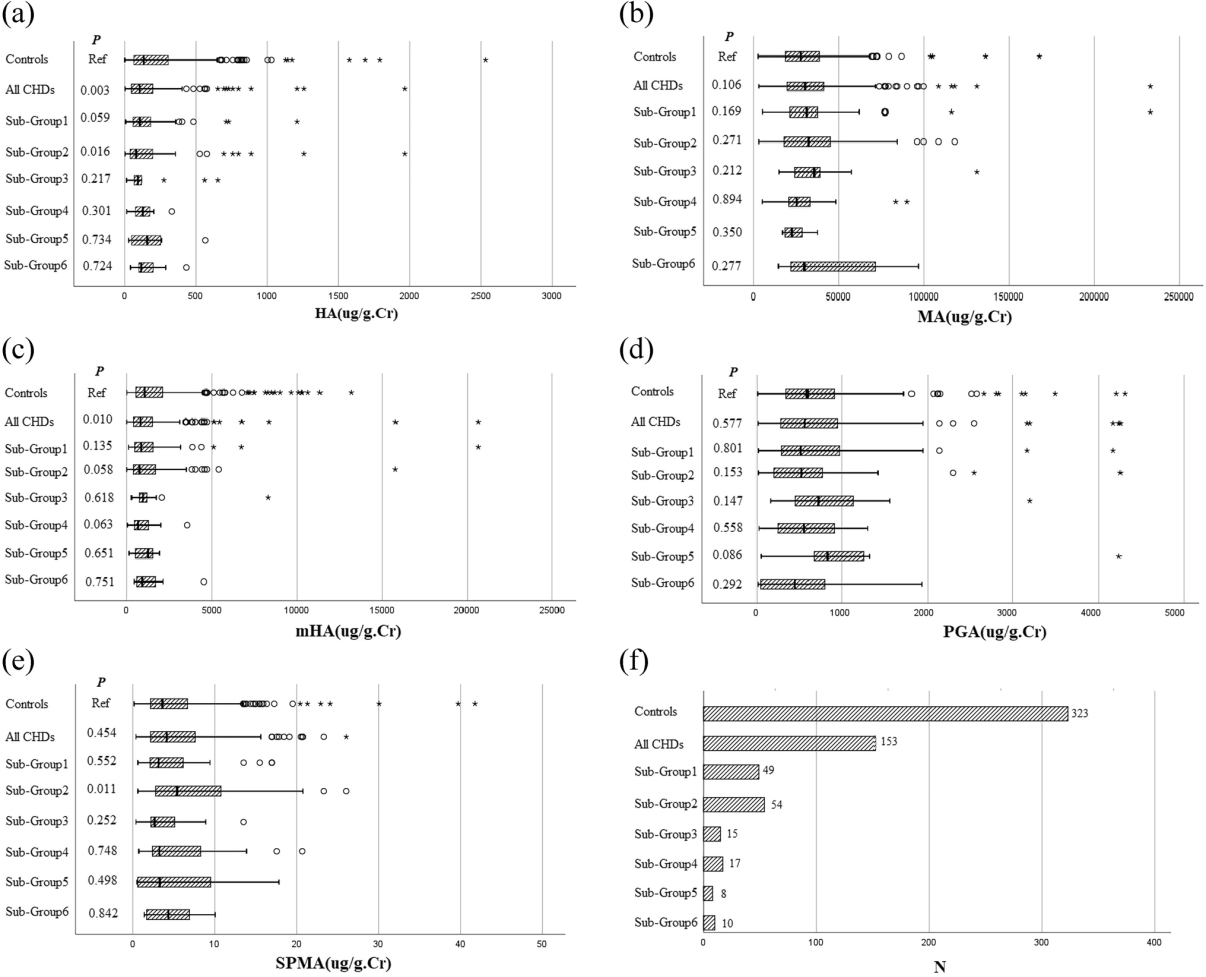
Concentrations of five urinary metabolites in the control group, CHDs, and subgroup ("○" inside the image represents mildly abnormal values (outside of Q1-1.5 times IQR or Q3+1.5 times IQR) and "*" indicates extreme abnormal values (outside of Q1-3 times IQR or Q3+3 times IQR))s

We compared the concentrations of MA, mHA, HA, PGA, and SPMA in the urine of mothers in different occupational groups and found no significant differences (see Fig. 1). We also compared whether the concentrations of these metabolites differed between the control group, all CHD groups, and the six CHD subgroups (see Fig. 2). The results showed that the concentrations of HA and mHA were lower in all CHD groups, and the concentrations of HA were also significantly lower in the left ventricular outflow tract obstruction group. In particular, we found that the concentrations of SPMA were significantly higher in the left ventricular outflow tract obstruction group (*p* = 0.011), but there were only 15 samples in this group. We also attempted to assess the relationship between SPMA concentrations and CHDs and found no significant association. It is important to note that our concentration values had outliers and that the subgroup sample size was small, and these findings need to be validated with a larger sample.

## Discussion

This multicenter case‒control study conducted in China found that environmental exposure factors such as the presence of public facilities near the maternal home, the use of or exposure to chemicals and hazardous substances during the perinatal period remained risk factors for CHDs in offspring after covariate adjustment. In particular, our data showed that all offspring whose mothers were engaged in agriculture production during the periconceptional period had CHDs, even though there were only 19 cases in total. Pregnant women in production manufacturing and related work may have an increased risk of CHDs in their offspring, particularly for conotruncal defects, left ventricular outflow tract obstructions, right ventricular outflow tract obstructions and abnormal pulmonary venous return. In addition, metabolite concentrations of benzene compounds in maternal urine were analyzed in this study, and the five metabolites did not differ between the case and control groups or among the different occupational types. This indicates that environmental exposure and occupational exposure to benzene compounds were not the main risk factors for CHDs in this study.

CHDs are highly prevalent problems with mostly unknown origins. One study found that multiple environmental exposures for pregnant women, including living in newly renovated rooms, living in homes near major traffic, smoking, and having a manual labor occupation, were significantly associated with CHDs in their offspring (Mamun et al. 2017). As shown in our study, both maternal living/working environment exposure and occupational exposure are risk factors for CHDs in offspring. Overall, the current evidence for maternal occupational exposure as a risk factor for congenital heart defects in offspring is inconclusive (Thulstrup and Bonde 2006).

Among the results of our study, we observed that the offspring of 19 pregnant women who were engaged in agricultural production all suffered from CHDs. We believe this is an important signal that exposure to harmful substances such as pesticides during engagement in agricultural production poses a great risk to the fetus. One study found that maternal involvement in agriculture or fishing was associated with a 79-fold increased risk of congenital heart defects, but the number of exposed groups was too small (Chia et al. 2004). Another study reported that exposure to pesticides during early pregnancy was associated with CHDs (Loffredo et al. 2001). These findings are similar to the findings of our study, but our sample only included 19 pregnant women who were engaged in agricultural production occupations, and the result needs a larger sample size to be validated. There are also studies with inconsistent findings: one study in the USA indicated that broad categories of pesticide exposure were not associated with CHDs but were associated with increased preponderance in specific CHD subtypes (Rocheleau et al. 2015). We found that pregnant women who engaged in production manufacturing and related work had a significantly associated risk in all four CHD subtypes, which also suggests that exposure to hazardous substances in this occupation may also increase the risk of CHDs in offspring. Therefore, further in-depth exposure studies for these types of occupations are needed. More definitive findings will allow for more specific protective guidance for pregnant women in the relevant occupations.

In our study, the sample size was too small, and the occupational classification was not divided into very detailed categories. It would be very valuable if the sample size were increased and some detailed subcategories of occupations could be analyzed in the future. This is similar to the US study on maternal occupation and major birth defects, with a detailed classification of occupations. The results show that conotruncal heart defects in the offspring of chemists and left ventricular outflow tract heart defects in the offspring of nurses showed positive associations (Lin et al. 2013). The results of the analysis for the occupational subcategories are more important for guiding the occupational protection of pregnant women.

Exposure to hazardous substances, such as organic solvents, is the most common hazard, including BTEXS exposure, and studies have shown that pregnant women exposed to organic solvents during pregnancy have an increased risk of giving birth to babies with CHDs compared to those who have not been exposed to organic solvents (Cordier et al. 1997). We searched the literature and found that very few studies have evaluated the association of BTEXS exposure during pregnancy with CHDs and CHD subtypes in offspring. A study conducted in Oklahoma evaluated the association between benzene exposure and congenital anomalies. However, their findings did not find an association between benzene exposure during pregnancy and the occurrence of CHDs in offspring (Janitz et al. 2018). This is similar to our results. The concentrations of the five metabolites measured in this study did not differ significantly between the CHD groups and the control groups. Our data also do not support the idea that exposure to benzene and its derivatives increases the risk of CHDs in offspring. Although the current data do not yet support a risk of benzene for fetal CHDs, BTEXS exposure is harmful, causing many congenital anomalies. A study published in 2021 found a significant association between high levels of phenylglyoxalate and a high risk of suburethral cleft in offspring by measuring phenylglyoxalate, a metabolite of styrene and ethylbenzene, in fetal meconium (Rouget et al. 2021). Another study found that exposure to BTEX during pregnancy in African American women is associated with the onset of inflammation in mid-gestation, which may be related to premature birth (Cassidy-Bushrow et al. 2021b). The results of a Spanish cohort study support the conclusion that maternal exposure to nitrogen dioxide and aromatic hydrocarbons, including BTEX, in early pregnancy can have adverse effects on fetal growth in mid-pregnancy (Aguilera et al. 2010).

We believe that the net effect of these metabolites on pregnant mothers is difficult to estimate accurately. Because the sources of contamination are multiple and multisourced, individual lifestyles as well as other factors may have confounding effects (Gorini et al. 2014). Our study found no differences in concentrations among occupations, which may be due to the relatively low or no exposure of Chinese women to benzenes at work, especially during pregnancy. It is necessary to note that the concentrations of these metabolites in our study are not a fully accurate representation of occupational BTEXS exposure. For example, xylene may originate from tobacco smoke exposure (De Jesús et al. 2020). Additionally, individual nonspecific metabolites do not necessarily represent the exposure concentration of benzene and its derivatives. For example, HA may be derived from benzoic acid in food.

### Strengths and limitations

The diagnosis of CHD (and subtypes) in this multicenter study is very clear and trustworthy. We observed statistically significant associations between certain environments and certain types of occupations during maternal pregnancy and CHD in the offspring, which can contribute to the understanding of risky exposures during pregnancy in the Chinese maternal population. However, there are limitations in the study. Firstly, We collected urine samples at the time of interview, i.e., after the onset of CHD. The pregnant women we recruited whose work and living environments were stable during pregnancy. Therefore, we assumed that the exposure to benzene compounds at the time of urine collection was essentially the same as the exposure during pregnancy., cohort studies should be conducted in the future to be more scientifically sound if possible in the future. Secondly, many baselines were different between the controls and cases in the study. Although these statistically different variables were adjusted as covariates in the regression analysis, these differences may cause confounding bias, which is a limitation of the design. Thirdly, the sample size was small, especially in the subtype group of CHDs. In addition, the concentration measurements had outliers and extreme values, which may have an impact on the statistical results. We hope to expand the sample size in future work to validate the results of this study.

## Conclusions

Living in a newly decorated house, having hazardous substance exposure, and having occupational exposure are suspicious influencing factors of CHDs. Our data suggested a higher risk of CHDs in the offspring of pregnant women who engaged in agricultural production and manufacturing. We measured the concentrations of five metabolites to determine the effects of occupational and environmental exposure to benzene compounds. However, for the time being, these data do not support an association between concentrations of metabolites of benzene compounds in the urine of pregnant women and CHDs in their offspring. Further epidemiological and toxicological studies are needed to examine and identify the causes of fetal CHD risk in pregnant women with agricultural production and manufacturing occupations.

## Supplementary Information

Below is the link to the electronic supplementary material.

Supplementary file1 (DOCX 23 KB)

## Data Availability

All available data are included in this published article.
