# Supplementary material for: Maternal environmental, occupational, and urinary metabolite levels of benzene compounds and their association with congenital heart diseases in offspring: a case‒control study in China
Source: Environ Sci Pollut Res Int. 2023 Apr 25;30(24):66021–32. doi: 10.1007/s11356-023-27015-z (PMC10182929; doi:10.1007/s11356-023-27015-z)
Supplement: Supplementary file 1 — Supplementary file1 (DOCX 23 KB) [file 11356_2023_27015_MOESM1_ESM.docx]

**Supplementary materials**

Table **S1**. Occupational type of the control group and all CHDs group.

| **Occupation code (major category)** | **Occupation (major category)** | **Control   n (%)** | **case  n (%)** | **Occupation code**  **(medium category)** | **Occupation**  **(medium category)** | **Control   n (%)** | **All CHDs  n (%)** |
| --- | --- | --- | --- | --- | --- | --- | --- |
| 2(GBM 20000) | Professional and technical staff | 153  (15.18) | 83  (10.29) | 2-02(GBM 20200) | Engineering Technicians | 12(1.19) | 5(0.62) |
|  |  |  |  | 2-05(GBM 20500) | Health Professionals and Technicians | 14(1.39) | 16(1.98) |
|  |  |  |  | 2-06(GBM 20600) | Economic and financial professionals | 77(7.64) | 32(3.97) |
|  |  |  |  | 2-08 (GBM 20800) | Teaching staff | 4(0.4) | 3(0.37) |
|  |  |  |  | 2-09 (GBM 20900) | Literature and art, sports professionals | 36(3.57) | 24(2.97) |
|  |  |  |  | 2-10(GBM 21000) | Press and publishing, cultural professionals | 7(0.69) | 3(0.37) |
|  |  |  |  | 2-99 (GBM 29900) | Other professional and technical personnel | 3(0.3) | 0(0) |
| 3 ( GBM 30000) | Clerical and related personnel | 182  (18.06) | 107  (13.26) | 3-02(GBM 30200) | Safety and firefighting personnel | 0(0) | 1(0.12) |
|  |  |  |  | 3-99 (GBM 39900) | Other clerical and related personnel | 182(18.06) | 106(13.14) |
| 4(GBM 40000) | Social production service and life service personnel | 492  (48.81) | 343  (42.5) | 4-01(GBM 40100) | Wholesale and retail service workers | 124(12.3) | 110(13.63) |
|  |  |  |  | 4-02(GBM 40200) | Transportation, storage and postal service personnel | 9(0.89) | 4(0.5) |
|  |  |  |  | 4-03(GBM 40300) | Accommodation and food service personnel | 7(0.69) | 26(3.22) |
|  |  |  |  | 4-04 (GBM 40400) | Information transmission, software and information technology services personnel | 9(0.89) | 5(0.62) |
|  |  |  |  | 4-05 (GBM 40500) | Full integration service personnel | 21(2.08) | 8(0.99) |
|  |  |  |  | 4-06 (GM 40600) | Real estate service personnel | 3(0.3) | 3(0.37) |
|  |  |  |  | 4-08 (GBM 40800) | Technical support service personnel | 8(0.79) | 8(0.99) |
|  |  |  |  | 4-09 (GBM 40900) | Water, environment and public facilities management services personnel | 1(0.1) | 1(0.12) |
|  |  |  |  | 4-10 (GBM 41000) | Residential service personnel | 285(28.27) | 151(18.71) |
|  |  |  |  | 4-12 (GBM 41200) | Repair and production service personnel | 1(0.1) | 1(0.12) |
|  |  |  |  | 4-14(GBM 41400) | Health service workers | 1(0.1) | 0(0) |
|  |  |  |  | 4-99(GBM 49900) | Other social production and life services workers | 23(2.28) | 26(3.22) |
| 5( GBM 50000) | Agriculture, forestry, animal husbandry, fishery production and supporting personnel | 0  (0) | 19  (2.35) | 5-01(GBM 50100) | Agricultural production personnel | 0(0) | 17(2.11) |
|  |  |  |  | 5-04(GBM 50400) | Fishery production personnel | 0(0) | 1(0.12) |
|  |  |  |  | 5-05(GBM 50500) | Agricultural, forestry, animal husbandry and fishery production support personnel | 0(0) | 1(0.12) |
| 6(GBM 60000) | Production manufacturing and related personnel | 30  (2.98) | 77  (9.54) | 6-02(GBM 60200) | Food and beverage production and processing personnel | 1(0.1) | 5(0.62) |
|  |  |  |  | 6-04 (GBM 60400) | Textiles, knitting, printing and dyeing personnel | 0(0) | 1(0.12) |
|  |  |  |  | 6-05(GBM 60500) | Textiles, clothing and leather, fur products processing and production personnel | 5(0.5) | 19(2.35) |
|  |  |  |  | 6-08 (GBM 60800) | Printing and recording media reproduction personnel | 0(0) | 1(0.12) |
|  |  |  |  | 6-09 (GBM 60900) | Education, industry, sports and entertainment products production personnel | 0(0) | 4(0.5) |
|  |  |  |  | 6-11 (GBM 61100) | Chemical materials and chemical products manufacturing personnel | 1(0.1) | 0(0) |
|  |  |  |  | 6-14 (GBM 61400) | Rubber and plastic products manufacturing personnel | 0(0) | 1(0.12) |
|  |  |  |  | 6-18 (GBM 61800) | Machinery manufacturing basic processing personnel | 0(0) | 2(0.25) |
|  |  |  |  | 6-19 (GBM 61900) | Metal products manufacturers | 2(0.2) | 2(0.25) |
|  |  |  |  | 6-24 (GBM 62400) | Electrical machinery and equipment manufacturers | 1(0.1) | 4(0.5) |
|  |  |  |  | 6-25 (GBM 62500) | Computer, communication and other electronic equipment manufacturing personnel | 7(0.69) | 14(1.73) |
|  |  |  |  | 6-28 (GBM 62800) | Electricity, heat, gas, water production and distribution personnel | 1(0.1) | 0(0) |
|  |  |  |  | 6-29 (GBM 62900) | Building construction personnel | 0(0) | 2(0.25) |
|  |  |  |  | 6-31(GBM 63100) | Production support personnel | 6(0.6) | 8(0.99) |
|  |  |  |  | 6-99 (GBM 69900) | Other manufacturing and related personnel | 6(0.6) | 14(1.73) |
| No coded | Not working or unemployed | 112  (11.11) | 118  (14.62) | — | — | — | — |
| Uncoded | Unidentifiable | 39  (3.87) | 60  (7.43) | — | — | — | — |
